# Designing Digital Mental Health Interventions to Meet the Needs of Older Adolescents: Qualitative Interview and Group Discussion Study

**DOI:** 10.2196/68950

**Published:** 2025-08-29

**Authors:** Rachel Kornfield, Sarah Alexandra Popowski, Emily Tack, Jack Svoboda, Miguel Herrera, Theresa Nguyen, Ashley Arehart Knapp, David Curtis Mohr, Jonah Meyerhoff

**Affiliations:** 1 Department of Preventive Medicine Northwestern University Chicago, IL United States; 2 Center for Behavioral Intervention Technologies Northwestern University Chicago, IL United States; 3 Department of Psychiatry and Behavioral Sciences Northwestern University Chicago, IL United States; 4 Mental Health America Alexandria, VA United States

**Keywords:** depression, anxiety, adolescents, qualitative data, SMS text messaging, digital mental health, user-centered design

## Abstract

**Background:**

Anxiety and depression are common in adolescents, but adolescents are often uninterested in formal mental health treatments or are unable to access them. Digital interventions can be delivered at scale to bridge critical gaps in mental health care but must address the needs and preferences of adolescents.

**Objective:**

This study aims to conduct qualitative research involving adolescents aged 18 years to inform both the design of digital mental health interventions for adolescents broadly and new features and refinements to incorporate in an automated SMS text messaging intervention, Small Steps SMS, that was originally designed for young adults.

**Methods:**

We recruited non–treatment-engaged older adolescents who were aged 18 years, lived in the United States, and had experienced depression or anxiety. In total, 12 participants were recruited through social media advertising and online self-screeners hosted by Mental Health America, a mental health advocacy organization. For 24 days, participants answered researcher prompts and engaged with one another in an asynchronous online discussion group, with a new discussion prompt released every 3 days. In parallel, partway through the discussion group, participants received interactive messages from Small Steps SMS, an automated SMS text messaging intervention that delivers daily dialogues supporting mental health self-management. Questions in the discussion group pertained to mental health challenges, help-seeking attitudes, perceptions of Small Steps SMS, and ways the program and other digital mental health interventions could meet the needs of older adolescents. A subset of participants (n=4, 33%) also completed interviews to elaborate on their responses. Thematic analysis was applied to transcripts of the discussion group and interviews to characterize user needs and design priorities when making Small Steps SMS and similar interventions available to adolescents.

**Results:**

Participants reported factors that contributed to their experience of mental health symptoms, including the transition from adolescence to adulthood, fears that the world is unstable and their futures are uncertain, and ineffective use of social media to cope with symptoms. Participants were proud of their generation’s mental health acceptance but also observed a generational divide in mental health stigma and literacy that could impede seeking help from parents and other adults. Participants appreciated that Small Steps SMS allowed them to pursue mental health self-management conveniently and independently. They suggested that the program and similar interventions address adolescent-specific challenges and facilitate intergenerational communication about mental health. They also recommended possible ways to increase engagement through peer-to-peer communication, gamification, and greater explanation of self-management strategies.

**Conclusions:**

Major life transitions affected adolescent participants’ mental health needs and preferences for digital mental health tools. While interactive automated messaging programs have the potential to support self-management in this population, program content and features should be adapted to adolescents’ needs.

## Introduction

### Background

Adolescence refers to the developmental stage between childhood and adulthood, defined by the World Health Organization as spanning ages 10 to 19 years [1]. Adolescence features unique biological developmental changes (ie, puberty), high attunement to social information, heightened emotionality, and evolution of social roles and self-concept [2-4]. These changes make adolescents susceptible to the emergence of mental health symptoms [5]. In addition, adolescent mental health has been affected by recent trends and events, including increases in screen time and social media use and the COVID-19 pandemic and related lockdowns [6,7]. Prevalence of past-year major depressive episodes among individuals aged between 12 and 17 years nearly doubled between 2005 and 2019, increasing from 8.7% to 15.7% and then further increased to 20.1% in 2021 [8-10]. In 2020, 20.5% of adolescents reported clinically elevated anxiety symptoms [11].

Despite high levels of mental health symptoms, few adolescents access mental health services. Typically, adolescents seek informal help from trusted friends and family members before turning to formal sources, such as medical professionals or school resources [12-14]. Among adolescents affected by recent major depressive episodes, only 40.6% received relevant treatment [10], and treatment rates for anxiety disorders were similarly low [15]. This reflects common barriers to help-seeking, such as stigma, which may be particularly salient to socially developing youth [4,16]. Other common barriers include costs, inadequate insurance, limited availability of services, low mental health literacy, negative beliefs about mental health services, and preferences for self-reliance [17-20]. Many states require parental consent for minors to pursue mental health treatment [21], which poses an additional barrier when adolescents anticipate or experience stigma from parents or guardians or prefer to pursue treatment independently [22,23].

There is a growing consensus that the unmet mental health needs of adolescents represent a public health crisis [24,25]. Untreated mental health symptoms in adolescence can lead to distress and increase the risk of poor academic performance, noncompletion of high school or college, psychiatric problems later in life, and suicide [26-29]. Early intervention can help adolescents manage symptoms and build coping skills, potentially changing the trajectory of mental health conditions into adulthood [30].

Digital mental health interventions (DMHIs) represent a potential solution to address the unmet mental health needs of adolescents. Most adolescents have access to and familiarity with digital devices, with 95% of US teenagers having access to a smartphone in 2022 [31]. DMHIs are generally well-received by adolescents, who report a preference for DMHIs that offer flexibility, self-reliance, opportunities for communication with others, and privacy [32]. Examples of existing DMHIs include mobile phone apps, therapeutic video games, and mental health–focused web programs [32,33]. These DMHIs have used many approaches, including coping skill education, mindfulness training, mental health coaching, self-monitoring and self-tracking, and social support [32,34,35]. Some DMHIs have been designed with scalability in mind, such as by delivering interventions in online spaces where adolescents seek mental health information and through communication channels that are already highly used by young people, such as messaging apps, texting, and social media [36-38].

Despite the potential alignment between DMHIs and the needs and preferences of adolescents, most DMHIs to date have focused on adults [39-41]. A systematic overview by Lehtimaki et al [32] concluded that DMHIs for adolescents demonstrate benefits for managing anxiety, depression, and stress when compared to nonactive control groups (receiving no treatment or wait-listed for treatment), but they did not find differences relative to active controls. They also found that completion rates among the reviewed interventions varied widely, ranging between 10% and 94%. Low engagement and high attrition are common issues in trials of DMHIs [35], and these issues are even more pronounced in the real world [42], reflecting the loss of structure and support outside of trials and a less motivated population of users [43]. To meaningfully address adolescents’ unmet mental health needs, there is a need for DMHIs that are effective as well as engaging in the context of adolescents’ day-to-day lives.

### Objectives

To ensure digital mental health tools have potential for real-world engagement and uptake, the digital mental health community increasingly uses human-centered design methods from the human-computer interaction field [43-45]. These methods engage end users to understand their needs, elicit their design ideas, gather iterative feedback on DMHI prototypes, and establish usability, with the goal of creating tools that are engaging and useful to real-world users.

Consistent with such a focus, this paper applies a qualitative human-centered design approach to understand how DMHIs can be designed and adapted to better address the needs and preferences of older adolescents who are not in formal mental health treatment. We focused on those not currently engaged in treatment to inform the design of DMHIs that can help close treatment gaps. We seek to provide insights into the mental health challenges faced by these older adolescents and their preferences concerning help seeking and self-management to understand how these needs and contexts might be addressed by design approaches and features for DMHIs. We solicit participants’ ideas for automated DMHIs broadly and for potential features and revisions to an existing interactive automated SMS text messaging intervention, Small Steps SMS, a program that was originally designed for young adults [46-48]. Automated interventions delivered via communication channels regularly used by young people (such as SMS text messaging) have the potential to expand access to mental health support on a large scale and at low cost. Alongside asking open-ended questions through an online discussion group, we deployed a 3-week version of the Small Steps SMS program as a technology probe [49], offering users experience of using a possible solution to generate ideas and allow specific feedback on the self-management content and interactions within the program. The program provides psychoeducation and supports applying several mental health self-management strategies that draw on evidence-based psychotherapies, such as cognitive behavioral therapy and acceptance and commitment therapy. Participants’ feedback will inform potential modifications to Small Steps SMS (eg, content revisions and new features) that can be prototyped and further assessed by end users.

## Methods

### Overview

To facilitate participation in study activities despite the sensitive and stigmatized nature of mental health issues, we used an asynchronous remote community (ARC) approach [50]. Originating in the human-computer interaction field, ARC allows participants to share their thoughts, opinions, experiences, and preferences anonymously within a private discussion forum and build on one another’s contributions. The ARC method seeks to build a comfortable context for active participation and ongoing interaction among participants that fosters a sense of community and collaborative ideation [51]. Recommendations emphasize groups that are relatively small (eg, 10-20 individuals) to facilitate rapport building and participation [52]. Participants may also complete independent activities to gain familiarity with technological solutions and prototypes, informing and deepening their responses within the ARC group [51]. In line with such an approach, participants in our study used the Small Steps SMS program beginning partway through the discussion group, receiving daily interactive SMS text messages. A subset of participants also completed semistructured phone interviews after the discussion group and the Small Steps SMS messaging concluded.

### Participants

All recruitment and study activities were fully remote, allowing individuals to engage from home or a location of their choice on their personal device. Participants in our study were recruited through paid advertisements targeting US-based users of social media platforms, namely Facebook (Meta Platforms, Inc) and Instagram (Meta Platforms, Inc), and through free self-screening surveys for depression and anxiety (ie, the Patient Health Questionnaire-9 [53] or Generalized Anxiety Disorder-7 scale [54]) hosted on the website of Mental Health America, a national mental health advocacy organization that serves largely young, non–treatment-seeking individuals with mental health symptoms [55]. The advertisement text described that participants could “Join a research study on technology and mental health.” Clicking the attached link led to a landing page with further details about the study and an option to continue to the eligibility survey. Potential participants who completed the eligibility survey were invited to enroll in our study if they self-reported being US residents, experiencing depression or anxiety (“Have you ever had an experience of depression or anxiety even if it was not diagnosed by a professional?”), having sufficient English language ability to participate in study activities, having access to a personal smartphone, and being aged 18 years. This focus on individuals aged 18 years allowed us to capture challenges around the transition from high school into early adulthood and to gather informed consent directly from participants who were legal adults. Exclusion criteria included being engaged in formal mental health treatment; visual, hearing, or motor impairments that would restrict individuals in completing study activities; self-reported serious mental illness (psychotic disorder or bipolar disorder); or suicidal ideation with plan and intent. Potential participants were required to provide a valid US mobile phone number on which they could receive SMS text messages. Individuals who completed screening were provided with a list of resources for accessing 24/7 mental health support, if needed (eg, suicide prevention hotline and crisis text line).

### Procedure

Data were collected between August 2023 and September 2023 through an online discussion group, parallel deployment of the Small Steps SMS program, and follow-up interviews.

#### Discussion Group

Participants were registered for an online discussion group based on ARC methods [50]. The discussion group was hosted on the FocusGroupIt [56] website. Participants were asked to submit text-based responses to prompts posted by the researchers every 3 days for 24 days (8 prompts in total). Prompts asked about participants’ mental health experiences, treatment and self-management needs, and perspectives on the design of digital tools for mental health (Multimedia Appendix 1). To encourage active dialogue and collaborative thinking, participants were also asked to reply to each other’s responses.

Responses in the discussion group were visible to all study participants; however, participants were identified to one another only by generic pseudonyms that were automatically assigned upon registration to the website (ie, Participant 1, Participant 2, etc), and under which all their comments were posted. Participants in the discussion group agreed to follow a code of conduct that advised them to engage others with respect and avoid disclosing personally identifying information or details about methods of suicide or self-harm. Responses were monitored daily for compliance with the code of conduct. Research staff were supervised by a member of the authorship team who is a licensed clinical psychologist. The team had a risk management protocol in place, such that a participant sharing any information signaling they were a risk to themselves or others would prompt a team member to contact the participant to administer risk assessments and provide resources, if needed. No such risks emerged; therefore, no follow-up was conducted. Text-based data from the discussion group were automatically saved by the website and downloaded by the researchers at the conclusion of the study.

#### Small Steps SMS Program

Participants in our study were also enrolled in a truncated version of the Small Steps SMS program following the third discussion group prompt. They received messages for 3 weeks. Within the group, participants were asked to reflect on their experiences with, impressions of, and suggestions for the Small Steps SMS program and similar programs.

**Figure 1:**
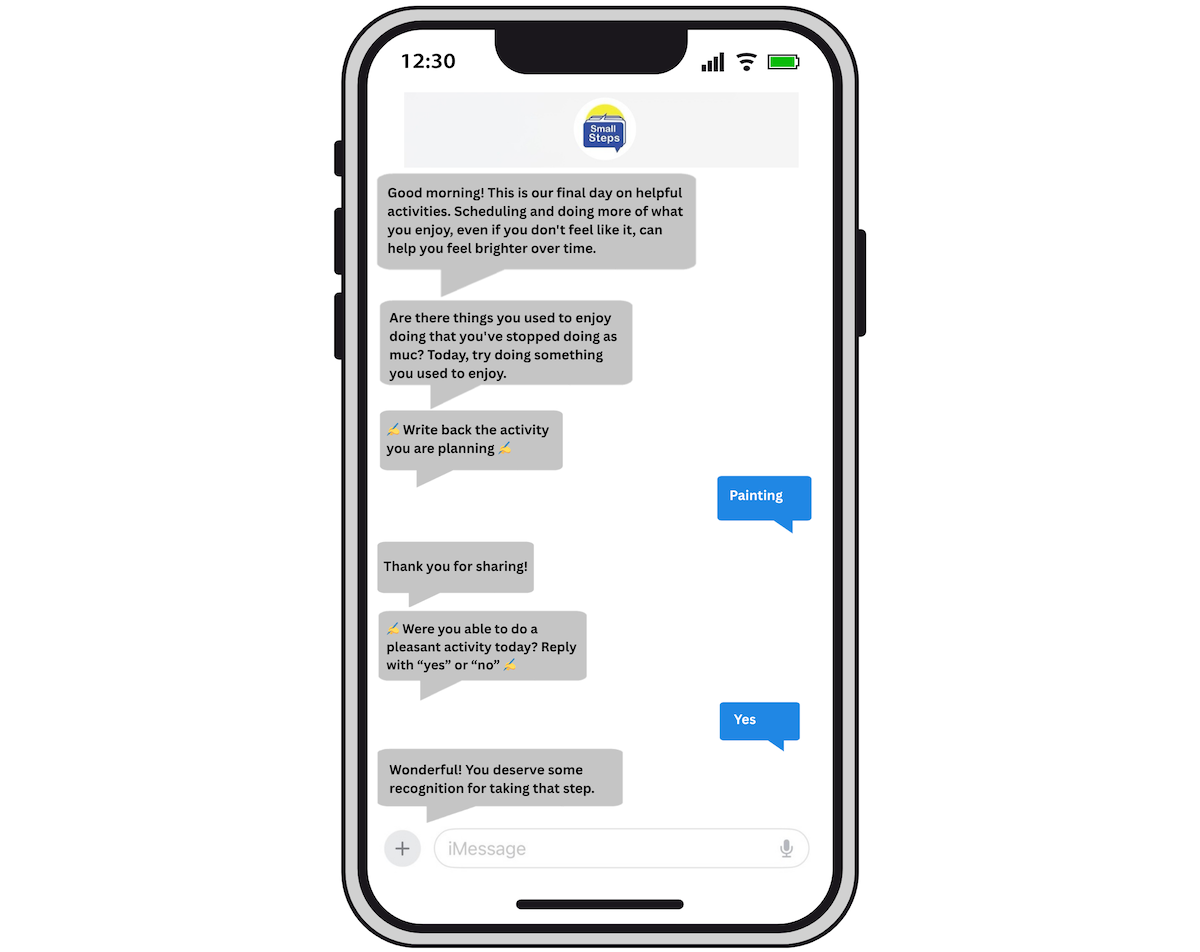
Example of interactive SMS text messaging dialogue with Small Steps SMS focused on behavioral activation.

**Figure 2:**
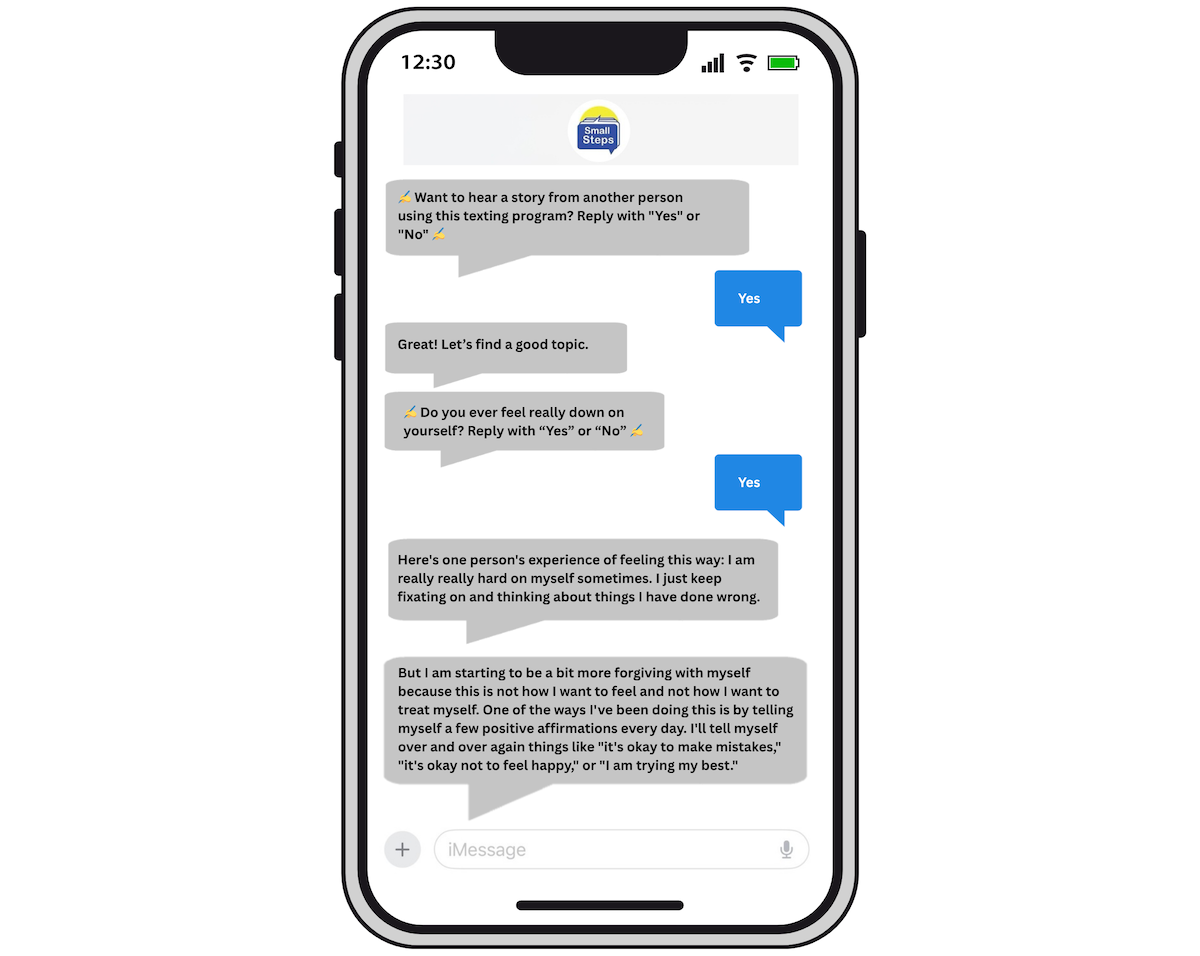
Example of interactive SMS text messaging dialogue with Small Steps SMS focused on self-compassion.

Small Steps SMS is an automated text messaging intervention to support self-management of depression and anxiety symptoms [46-48,57]. Small Steps SMS was designed through a human-centered design process encompassing elicitation activities, co-design workshops, prototype usability testing, and interviews [46-48], leading to a focus on sustaining engagement through (1) delivering diverse interaction types (eg, peer stories, reflection questions, psychoeducation, and action prompts) and (2) supporting low-stake experimentation with a variety of evidence-based self-management strategies (eg, thought challenging, behavioral activation, valued living, and self-compassion). Dialogues are interactive, with a subset of messages asking participants to respond with their feedback, choices, and experiences. Figures 1 and 2 show example interactions with Small Steps SMS. The number of daily messages sent to each participant was dependent on user responses, such that participants who were more engaged with the program received more messages. Participants were not required to respond to messages from Small Steps SMS and received no compensation for engaging with the program. They could send the word “stop” at any time to end all messages. Responses to the program were monitored daily for indications of safety risks. No such risks emerged, and no follow-up was conducted.

#### Interviews

Participants were invited to complete optional semistructured interviews with researchers after discussion group activities concluded. The main purpose of the interviews was to allow participants to share detailed feedback on the researcher prompts and Small Steps SMS that may not have been covered in the discussion group responses. Interviews lasted approximately 20 to 25 minutes and were conducted via the Zoom teleconferencing platform (Zoom Communications, Inc). Participants could turn their video on or off based on their preference. Interviews were audio recorded and transcribed.

### Ethical Considerations

All study procedures were approved by the Institutional Review Board at Northwestern University (STU00211168). Participants provided informed consent before the commencement of study activities. They were informed that they could skip any questions they liked or end their participation. All study data were deidentified before analysis. Participants were compensated for research activities. In the discussion group, responding to each researcher-posted discussion prompt was compensated with US $7. For each prompt, responding to at least one other participant was compensated with US $2. Thus, participants could earn up to US $9 per prompt and US $72 total for participating in the discussion group, which was delivered in the form of a gift card upon completion of the study. Participants could earn an additional US $8 to complete an interview (in the form of a gift card).

### Data Analysis

Deidentified transcribed data from the discussion group and interviews were subjected to thematic analysis [58]. Such an approach allows for inductive identification of themes and corresponds to the lack of established theory related to the research topic [59]. The lead author, who is an experienced mixed methods researcher, trained the other 3 coders, who were research staff, in qualitative methods. These 4 coders, who are also authors of this paper, first became familiar with the data by reading the discussion group and interview transcripts. They performed open coding to identify preliminary codes and met to discuss and prioritize these codes, guided by each code’s relevance to the research questions. Prioritized codes and their definitions were captured in a shared codebook. The coders then applied the codebook to overlapping transcripts using the qualitative data analysis software Dedoose (SocioCultural Research Consultants, LLC) and subsequently met to discuss and resolve coding discrepancies and refine and consolidate the codebook. Approximately 4 overlapping coding rounds were completed before discussions ceased to yield codebook revisions. Three of the coders then divided the uncoded transcripts and applied the final codebook. Key themes were identified that encompassed the coded data; these were discussed and refined with the larger authorship team, and excerpts were selected to illustrate each theme.

## Results

### Overview

In total, 47 individuals initiated the screening process. Of these, 22 (47%) individuals were excluded because they did not meet 1 or more of the eligibility criteria. Excluded individuals were not aged 18 years (n=11, 50%), were in mental health treatment (n=8, 36%), reported serious mental illness (n=6, 27%), or reported severe suicidality (n=2, 9%). In total, 4 (9%) of the 47 individuals did not complete the screening process. A total of 21 (45%) individuals were deemed eligible and invited to read and sign the consent form, of whom 17 (81%) did. Of these 17 individuals, 14 (82%) completed the required steps to enroll in our study, and, of these individuals, 12 (86%) engaged in the study’s discussion group and are included in our analyses.

Table 1 presents participant demographics, treatment history, and symptom severity. Participants were mostly women, were racially and ethnically diverse, and half (6/12, 50%) of them reported previous mental health treatment. The current symptoms of both anxiety and depression were moderate, on average.

**Table 1:** Demographics, mental health history, and symptom levels of participants aged 18 years included in qualitative human-centered design activities to improve digital mental health interventions.

Characteristic			Discussion group (n=12)		Interview (n=4)
**Gender, n (%)**					
	Woman	8 (67)		3 (75)	
	Man	3 (25)		1 (25)	
	Nonbinary	1 (8)		0 (0)	
**Race, n (%)**					
	African American	3 (25)		2 (50)	
	Asian	3 (25)		1 (25)	
	White	3 (25)		1 (25)	
	Multiracial	3 (25)		0 (0)	
**Ethnicity, n (%)**					
	Hispanic or Latinx	4 (33)		1 (25)	
	Not Hispanic or Latinx	8 (67)		3 (75)	
**Mental health treatment history, n (%)**					
	Previously consulted a mental health professional	6 (50)		1 (25)	
	Previously received a prescription for a mental health condition	3 (25)		1 (25)	
**Depression and anxiety symptom scores, mean (SD)**					
	PHQ-9^a^	13.42 (4.25)		13.00 (5.60)	
	GAD-7^b^	12.83 (3.79)		13.25 (5.06)	

^a^PHQ-9: 9-item Patient Health Questionnaire.

^b^GAD-7: 7-item Generalized Anxiety Disorder.

On average, participants responded at least once to 5.92 (SD 2.43) of the 8 researcher-posted prompts. They responded an average of 18.08 (SD 11.54) times (including direct responses to prompts and replies to one another), contributing an average of 1238 (SD 839.79) words across all responses.

Our study included adolescents with any previous experiences of depression or anxiety; however, most participants reported mental health symptoms that were current. Participants’ symptoms varied in duration; some had experienced chronic and persistent symptoms lasting months or years, while for others, symptoms had only manifested recently. Participants identified several key factors that they associated with their mental health challenges, including the life transition from adolescence to young adulthood, uncertainty in their own future, and the belief that the world is unstable. Participants also shared their perspectives on the influence of technology in mental health, including how technology can be both helpful and harmful for mental health, and its impact on mental health literacy and stigma. Finally, participants shared their perspectives on DMHIs and how these tools, including Small Steps SMS, can be optimized to address the specific mental health challenges they face and to better engage them.

### Factors Influencing Mental Health

Participants reported experiencing a major life transition in late adolescence and shared that certain factors encountered during this transition may have contributed to the worsening of their mental health. One factor highlighted was the pressure to manage an overwhelming number of tasks, all at once, which included school-related responsibilities, commitments to extracurriculars, and employment. This was described by one participant as follows:

I feel like things that affect this generation is like stress, mainly I feel like there is a lot of expectations being pushed. And you try to do your very best, and sometimes people want to work, but we’re also expected to do that. And sometimes you have extracurriculars and so, you have to do all that.

P6

Participants also reported feeling overwhelmed by a growing number of responsibilities after graduating high school and transitioning to college, as stated by one participant as follows:

So, coming to college is a really big culture shock because you’re so—you’re responsible for everything. Everything’s on you now. Everything bad that happens, you have to ask yourself, oh, was it my fault? Did I miss something?

P7

Participants indicated feeling scared, exhausted, and unmotivated in response to changes in their daily routines, which they perceived as rapid and drastic. Overall, despite acknowledging that the transition from adolescence to adulthood is characterized by both positive and negative aspects, they described their own experiences to be jarring. One participant reflected as follows:

I graduated this summer and now that it’s almost fall I’m feeling weird about how life is going to be so different now because it was basically the same thing for so many years of my life. I wish I had more time to adjust to that before I’m shoved into “real life.” It’s kinda scary to be honest.

P1

Challenges in transitioning from adolescence to adulthood also extended to those who did not enroll in college:

One of the biggest challenges I’m currently facing is with moving out to live on my own and whether or not I want to move on to college.

P2

Feeling socially excluded during the transition from adolescence to adulthood was identified as a significant trigger for symptoms of depression and anxiety, and many participants reported struggling with these types of social challenges:

The biggest challenge in my life is adjusting to university with a very much lively social scene I’m not used to. It’s hard adapting right now where everyone seemingly already have found their crowd of people to rely on.

P11

Similarly, a participant considered themself to have greater social challenges than their peers, partly because they were a commuter student. They shared the following:

I feel pretty left out while everyone seems to be getting together really well. And that spiralled into me being sad and replaying a bunch of moments from last year that I still haven’t moved on from yet...I start feeling out of place, feeling very tired, and my body feels very irritable.

P5

Many participants also identified uncertainty about the future as a factor that contributed to their experiences of anxiety and depression. Participants worried about their ability to successfully manage both short- and long-term goals:

The biggest thing in life that’s making me depressed and anxious is my future. I’m worried about what if I can’t make it through my courses and I fail. Will I be able to make a living?...Will I ever be able to actually find someone with the way dating culture is these days? Basically everything about the future and it’s exhausting. I’d just like to be able to live my life in the moment and enjoy it instead of constantly worrying about basically everything and not feeling so tired and not like doing anything.

P1

Finally, participants identified the belief that they were coming of age in an unpredictable world as a contributor to their mental health issues. For example, one participant shared the following:

With the state of the world these days, it’s really hard to think positively about the future.

P9

Participants’ fears that the world is unpredictable were based, in part, on their experiences of growing up during the COVID-19 pandemic. They believed the pandemic had long-lasting effects on their mental health, disrupting the transition from adolescence to adulthood, which was explained by one participant as follows:

...a lot of people in this young generation are stuck in their pre pandemic life where they were carefree and dependent on their parents. Counseling therapy should consider the fact that the teens from this generation need time to adjust to their new age and responsibilities.

P8

Overall, participants associated the transition from adolescence to adulthood with the experience of depressive and anxious symptoms. These challenges were further exacerbated by participants’ perceptions of an unpredictable world and an uncertain future.

### Perceptions of the Role of Technology in Mental Health

#### Overview

Participants perceived technology to be deeply embedded in their daily lives and often described it as a source of entertainment and distraction. For many participants, technology offered an escape from daily stressors and was considered a useful coping tool; however, some relayed that overreliance on technology could reinforce negative habits (eg, self-isolation) and worsen mental health symptoms. Despite reporting ambivalence regarding the impact of technology on mental health, participants believed that technology played a significant role in enhancing mental health awareness and reducing stigma, particularly within their own generation.

#### The Dual Impact of Technology on Mental Health: Helpful and Harmful

Participants reported relying on technology as a coping tool when dealing with acute mental health challenges. Their digital devices were described as a means of escape when they were feeling mentally unwell, allowing them to seek social support and connection. One participant shared the following:

Technology connects us to people all over the world and gives people a sense a community, which I think is important for mental health or even just for having people to reach out to when you need them.

P2

Most participants discussed having used the internet as a tool to learn more about their mental health symptoms and others’ experiences. Some had also disclosed their experiences of depression and anxiety online to others, sought to understand available treatment and support options, or used mobile phone apps for mental health and well-being.

Some individuals also reported that social media, specifically, served as a useful coping tool for social anxiety, and attributed its usefulness to social norms that sanction the use of cell phones in public. One participant described the following:

It’s a good distraction sometimes when you’re, I guess, alone. Like, let’s say you’re in a public space, and you feel alone. You can just go on social media and just be in your own pot, and just scroll through it or whatever.

P4

Many participants recognized that technology has a dual impact on mental health, noting that it has the potential to not only improve but also worsen symptoms of depression and anxiety. Participants reported beliefs that using technology as a mental health coping tool could encourage avoidant behaviors and potentially exacerbate symptoms. P10 summarized this concern, sharing that they frequently turned to technology to escape their problems, but technology did not address the root of these problems. They summarized that technology “hurts me more than it helps, I end up spending too much time on reels and ignoring the issues completely.”

Similarly, one participant described the following:

I think I fell victim to using technology as a distraction all the time. But even I know that technology, like social media doesn’t fix everything and can even make it feel worse.

P7

One participant also questioned whether using technology in place of traditional social engagement could have unintended negative consequences:

But then, on the other side, I think it can be very harmful because of the things that people use technology for. Social media can be very harmful. And I feel like some people can use technology as a way to avoid people.

P7

While participants recognized that technology could temporarily help them escape their problems, they recognized its potential to serve as an unhealthy coping mechanism that could prevent them from addressing the root causes of their mental health issues and engaging in more effective treatment strategies.

#### Reducing Mental Health Stigma Using Technology

Participants strongly advocated for mental health awareness and resources, emphasizing that mental health concerns should be taken seriously and that access to treatment should be readily available. They reported observing a generational gap in mental health awareness, believing that younger generations possess a greater understanding of mental health issues compared to older generations. One participant shared the following:

I think mental health issues are so normalized with younger people compared to older people...I think younger people are more understanding and open to talking about mental health issues because they’ve been exposed to it more often.

P7

Participants attributed generational differences in mental health awareness largely to the rise of the internet, and specifically social media, stated by one participant as follows:

People my age grew up with social media and the Internet as it was forming, so naturally we were a lot more exposed to mental health issues (both good and bad) than those who are older.

P2

Thus, many participants felt that technology had benefited their generation by raising mental health awareness, which enabled them to openly discuss mental health issues with others and seek help when needed; however, many participants reported fears that their mental health issues would be dismissed or invalidated by members of older generations. One participant described the following:

Older generations, in my experience, tend to brush off any negative emotions or feelings as being weak or made up for attention. This has to do with the lack of research and widespread knowledge of mental health issues at the time. So, now, we see a lot of older people tend to say they’re fake simply because it’s newer information, and they still need time to adjust to it being the norm.

P9

Similarly, a participant stated the following:

Younger people may just be told they’re being dramatic or oversensitive or to just get over it or deal. Or that it will pass, you’ll grow out of it etc.

P1

Lower mental health awareness among older generations was perceived to be a barrier to mental health treatment by participants, in part because adolescents often must go through a parent, teacher, or other adult to access mental health resources. One participant shared some of their concerns about discussing mental health treatment with a parent:

Well, personally, I would feel uncomfortable because my dad would be like, why are you doing that? What do you need it for? He’s just gonna ask a lot of questions that I’m just gonna pause it, and don’t know the answer to any of it.

P4

Some participants viewed technology as an opportunity to bridge gaps in mental health awareness and facilitate intergenerational conversations on mental health topics; however, those who lacked confidence in involving adults in their mental health decision-making were more likely to report an interest in seeking resources independently. Overall, participants were interested in mental health solutions that could help them treat symptoms of depression and anxiety without stigma or judgment. Participants’ preferences for how a digital tool might meet their needs are described in the next section.

### SMS Text Messaging as a Tool for Mental Health Support

#### Endorsement of the Program

Adolescents in our study expressed interest in digital tools for mental health self-management, and most engaged actively with the Small Steps SMS program. All participants responded at least once to the program, with the average number of responses over the 3-week program being 28.66 (SD 25.71).

In the discussion group, participants shared that they perceived the Small Steps SMS program positively, describing it as convenient, accessible, and familiar due to its use of SMS text messaging. One participant explained as follows:

I definitely think the fact that it’s a texting program is a factor that would make people my age sign up because it’s low pressure and easy to fit in when you can.

P1

Participants believed that Small Steps SMS could be easily integrated within their daily routine because they could engage with it on their own time and in their own space. Others shared that SMS text messaging reduced some of the anxiety and vulnerability that can arise face-to-face. One participant stated the following:

It also feels like there’s much less pressure because it’s a texting program and not face to face/physically talking to someone.

P3

One participant also provided their perspective on the sense of privacy that texting provides:

I think a text service would be very helpful for when people are in public and they’re dealing with a mental health issue or if they are in places where they don’t want their mental health to be aired out for everyone to see.

P7

#### Addressing Adolescent-Specific Issues in Program Content

While participants held favorable perceptions of Small Steps SMS, they believed that the program could be improved for older adolescents. Participants thought that Small Steps SMS and other similar programs should tailor their content to the major life changes that their age group faces, which they had described in response to earlier prompts (eg, including the transition to college, moving away from home, or joining the workforce). One participant stated the following:

...adding more content regarding the life changes some people in the group might be going through and how to get through the mental barriers they are going through with the changes.

P12

Similarly, one participant emphasized the importance of including content tailored to the transition to college, which they identified as a particularly challenging life change:

I think it would be really helpful, especially in the first few months of college, when you’re still trying to get to know people. You’re still trying to figure out how to be an adult, how to be on your own, how to deal with homesickness, and stuff like that.

P7

Aside from major life transitions, some participants highlighted how parents, teachers, and other adults can impact their ability to seek help for mental health symptoms and advocated for a program that could help them navigate these intergenerational challenges. For example, one participant imagined a program that could help younger generations communicate their mental health needs to older generations:

For people my age, I feel like it would be more geared towards helping them find support and being comfortable talking about it with their parents.

P12

#### Reimagining Engagement Strategies for Adolescent Users

In addition to adapting the content within SMS text messages, participants identified ways that Small Steps SMS and related programs could improve the user experience and increase engagement among adolescents. These suggestions included gamification, opportunities for peer-to-peer communication, and more scaffolding of self-management strategies.

Several participants thought that Small Steps SMS and similar self-management interventions could benefit from the inclusion of game-like components. For example, one participant stated the following:

I think a fun and interactive way to make a tool for people my age (if I had the skill to make it ha ha) would be a mobile game where you do tasks to get points and earn rewards...After a certain amount of points, you get to pick a charity to donate some money to. It could also do e-gift cards as rewards as well.

P3

Participants theorized that the addition of more tangible rewards would increase engagement among adolescents. One participant provided additional guidance on how these types of rewards could be incorporated within programs that suggest daily self-management tasks:

I think a tool that people my age would like would be an app or game that interests them and gives them in-game rewards for different things they do. This would be able to ask them different prompts about how they’re feeling while not making it all about mental health. With this, it can give them the opportunity to be honest with how they’re feeling while also making sure that the thing that they’re using isn’t fully about how they’re feeling.

P12

Overall, the possible addition of gamified elements was identified as an opportunity for DMHIs to incorporate an element of fun and allow individuals to see beyond their mental health issues while also addressing symptoms.

Moreover, participants thought the program could benefit from the addition of peer-to-peer communication. Many participants indicated a strong interest in communicating with other adolescents who experienced similar challenges. One participant explained the following:

I think that people my age would like being in a program like Small Steps because they can talk with people near their age going through the same life changes as they are and can experience it together and we can help each other through the mental health part of things.

P12

Participants particularly valued opportunities to connect over similar hobbies and interests. They saw these connections as a pathway to build community and reduce the stigma of mental health issues.

Finally, some participants thought that digital self-management interventions, such as Small Steps SMS, should incorporate more technical guidance as well as detailed instructions that break down how to use each psychological strategy introduced in the content. One participant stated the following:

[Mental health interventions] should have an easy-to-use interface with clear directions on how to use it...I’d add in videos or something that would give step by step guides to some of the techniques.

P1

Similarly, a participant shared the following:

I agree with adding more direction to the messages. I feel the messages could be a bit more substantial.

P6

Thus, presenting program content alongside gamified tasks, integrating peer-to-peer communication, and greater support for learning psychological strategies were seen as ways to increase engagement and satisfaction among these older adolescents. Participants noted that such adaptations could improve Small Steps SMS and also endorsed such strategies as broadly applicable to a range of automated DMHIs that support adolescents in self-managing mental health symptoms.

## Discussion

### Principal Findings

This study sought to understand how to design and adapt DMHIs to address the mental health needs identified by older adolescent participants. We focus on automated interventions, including the Small Steps SMS program, which delivers self-management support through daily interactive SMS text messages. Through an online discussion group and series of interviews, non–treatment-engaged participants aged 18 years identified key mental health challenges and barriers to effective coping that are tied to their transition from adolescence to young adulthood and discussed ways that DMHIs could address them. The transition from high school to college or the workforce was regarded as highly influential for participants’ mental health. They also viewed their mental health symptoms as related not only to being young but also to being young in the present moment, pointing to the perception that the world is becoming increasingly unstable and inhospitable. They highlighted the complex influence of technology and social media on young people, contending that the impact of these technologies on an individual’s mental health depends on how they are used, making them either beneficial or harmful. As a potential solution to address their mental health concerns, participants endorsed Small Steps SMS as helpful, describing its automated approach to be a nonstigmatizing and low-burden way to learn self-management strategies while avoiding disclosure and possible dismissive reactions from parents and other adults. SMS text messaging (as a medium) was viewed as convenient and familiar. They suggested several changes to Small Steps SMS and considerations for DMHIs generally, highlighting that these tools should address the specific challenges faced by older adolescents, including the disruption while transitioning out of high school and navigating intergenerational conversations about mental health. They also thought that programs could be structured to further engage them through game-like elements, peer-to-peer contact, and more detailed guidance on self-management practices.

### Comparison With Prior Work

Given the extent of unmet mental health needs in the United States across demographic groups, many DMHIs are designed to be suitable across various individuals in need [39-41]; however, it is increasingly recognized that programs may be more appealing and potentially more effective when designed for narrower segments of the population [60,61]. This can be achieved either through programs that are targeted (ie, making them available to certain segments of the population) or tailored (ie, where content or components are delivered based on users’ assessed characteristics) [61]. When targeting and tailoring DMHIs, designers must weigh the potential benefits of focusing on particular constituent groups against the resources required to do so [62]. Past work has developed programs aligned with the needs of racial and ethnic minoritized groups [61], those with limited resources or specific challenges (eg, homelessness and trauma) [63,64], and age ranges, including some that focus on adolescents [32]. Subsequently, we interpret our findings in relation to the larger body of work on the mental health challenges faced by adolescents and discuss approaches to adapting digital tools to address these challenges.

For young people, past work has focused on developing programs that reflect their distinct mental health needs, including through considerations of mental health literacy, differences in technology use, and settings for disseminating and implementing digital mental health tools (eg, schools, pediatric care, or public services such as libraries) [41,65,66]. Our findings are consistent with this work in suggesting that our participants wanted program content to address the specific concerns and challenges faced by their age group. Participants particularly wanted validation and guidance related to the transition from adolescence to adulthood, which was experienced as highly overwhelming and disruptive to their mental health. Corresponding to this finding, previous literature on late adolescence has also described rapid and disorienting transitions in social standing and from familial dependence to independence [3,67].

Furthermore, participants wanted tools that they could access independently, partly reflecting their perception of older generations as less aware and accepting of mental health challenges. Participants often thought that their mental health problems would be trivialized because of their age and avoided help-seeking through parents or other adults of older generations. Relatedly, some recent work has proposed a “prevalence inflation hypothesis,” wherein exposure to well-intended mental health awareness campaigns on social media and in schools and other settings could drive young people’s inaccurate self-diagnoses or potentially exacerbate their symptoms [68]. While our findings cannot speak to the validity of this hypothesis, they suggest that our adolescent participants perceive skepticism about their mental health concerns to be widespread, and they find such views invalidating and stigmatizing. To avoid unsupportive responses to mental health disclosures, they were interested in accessing treatment on their own, although some also wanted support in raising or discussing mental health issues with parents or guardians. Some related work has explored technology as a tool for brokering intergenerational communication, including facilitating parent-child shared experiences, conversations about health topics or emotions between parents and children, in-the-moment parenting support, and self-monitoring family functioning [69,70]. Opportunities may also include dyadic interventions (ie, those that help the adolescent while also offering parallel guidance for the parent in how to support their child with a mental health issue), providing opportunities to rehearse mental health disclosure and build communication skills, and educational approaches that target parents’ mental health stigma.

Our findings also suggest that these participants see their generation’s acceptance of mental health issues as an important strength. In this regard, strength-based interventions, which are conceived to emphasize and understand the experiences, values, perspectives, and strengths of systemically marginalized people [71], may be useful for adolescents (who tend to identify closely with their generation). Participants noted that they are united not just as young people but as young people coming of age after the COVID-19 pandemic who are witnessing historic levels of social and technological change as they enter adulthood. Some strength-based interventions focus on individuals’ strengths, such as one digital tool designed for transition-age youth on the autism spectrum that supports users in identifying their strengths and expressing those strengths to others [72]. Other strength-based approaches can build an empowering collective narrative (eg, around experiences as refugees) [73]. Helping adolescents claim and take pride in shared traits, such as openness and acceptance, may be a way to support adolescents through a sense of community and collective action. This may be particularly important given that participants felt their age group was misunderstood and disparaged.

Our data also speak to the deep enmeshment of technology in participants’ daily lives and the dual role of technology in mental health. Potential negative effects of technology on the mental health of adolescents have been of great public concern, with the US Surgeon General recently issuing a warning about the negative effects of social media use in adolescence [74]. The extent and nature of these effects have been debated, as the research literature has yielded complex and sometimes contradictory findings [75-77]. However, less work has examined how adolescents themselves perceive and balance the potential benefits and harms of technology. Our findings suggest considerable nuance in how these older adolescent participants think about and use technology. Among our participants, technology was often used as a coping tool, sometimes successfully. Many described how much they had benefited from the social awareness and destigmatization that the internet had fostered related to mental health, and their regular use of mobile phones was key to achieving continuous reach of DMHIs, facilitating engagement in mental health self-management. However, they also recognized important harms of technology, particularly social media. Often used to cope with mental health symptoms, this use could backfire and lead to loss of productivity, avoidance, and isolation. Importantly, participants did not view all uses of technology as harmful but acknowledged variability across types of technology (eg, social media vs texting) and motivations for use (eg, avoidance vs seeking mental health information). This is consistent with past work describing distinctions between passive use of social media (eg, browsing feeds), which may have negative effects on mental health, whereas more active and targeted use (eg, direct communication with close ties) may have benefits [78,79]. Participants drew on their deep experience using apps and social media sites to suggest engagement strategies that might better sustain their use of DMHIs, emphasizing peer-to-peer communication and gamification—approaches which have also been endorsed by adolescents in previous literature [32,33,80].

### Implications for Automated Messaging Interventions

These findings suggest specific modifications and new features for Small Steps SMS and related DMHIs. Participants’ feedback suggests that interactive SMS text messaging is a promising pathway to reach those in their age range and also that content should be extended and refined, and new engagement strategies should be considered. As far as content, programs for older adolescents could be refined to directly address topics, such as the disruptions in completing high school and moving on to college or the workforce, intergenerational conversations about mental health, and redefining one’s relationship with social media. Adaptations to Small Steps SMS may include new modules or tailored examples and information when introducing psychological strategies (eg, existing “help-seeking” content could be expanded to provide guidance on approaching parents for mental health support). As participants valued peer-generated content, these issues may also be addressed through crowdsourced peer stories from other adolescents showing how they used a psychological strategy to address common challenges such as isolation, feeling overwhelmed, or invalidation. Participants also suggested new features for Small Steps SMS and related DMHIs. Some wanted detailed, step-by-step instructions on using psychological strategies, which might take the form of supplementary interactive guidance that can be launched at the user’s request (eg, through a text-based command such as “example” or “expand”). Others highlighted direct peer-to-peer communication, which could be facilitated through a web-based asynchronous discussion group parallel to the texting program or potentially through the program connecting peers to one another for direct text chat, as has been used in other texting interventions [81]. Participants also suggested game-like elements. In the context of Small Steps SMS, gamification could involve adding points or other rewards that can be collected by completing the daily tasks or activities suggested by the program or achieving streaks (eg, completing 3 behavioral activation tasks over 3 days). Before evaluating adolescent-targeted versions of the program, new content and prototypes of new features can be refined through feedback from users as part of our ongoing human-centered design process.

### Limitations

Adolescence spans an age range from 10 to 19 years [1], and adolescents typically progress through profound changes in physical maturity, social roles, sense of self and identity, and emotional and intellectual development [2-5,67,82]. Our study focused on a relatively narrow subgroup of older adolescents—those who are aged 18 years. We prioritized direct access to participants who are legal adults because parental involvement (eg, parental consent) can act as a barrier to help-seeking and potentially to mental health research participation [22,23]. Consequently, our study is limited in what we can conclude about the needs of younger adolescents. While we asked participants to reflect on their recent experiences in high school and comment on how younger adolescents may receive the Small Steps SMS program, we note the importance of further research prioritizing those aged <18 years.

In addition, although sample sizes are often small in human-centered design work [83] and small group sizes for online discussion groups are recommended to increase participants’ comfort and engagement [84], we acknowledge that using additional methods and recruiting a larger sample may be helpful to triangulate findings related to the mental health challenges adolescents face and their relationship with technology. Our qualitative findings can suggest potential hypotheses and theories to be evaluated using quantitative methods [59]. Finally, while we recruited a diverse group of participants in regard to race and ethnicity, their recruitment online and the study’s focus on technology-based solutions may have contributed to overrepresenting those with interest in and access to technologies. Given the study’s emphasis on SMS text messaging and the exposure to the Small Steps SMS program, it is also possible that participants were predisposed to come up with messaging-related solutions at the expense of other design directions.

### Conclusions

Adolescents experience high levels of mental health issues but have limited access to treatment. We engaged older adolescents to better understand how to design digital mental health tools to address their mental health challenges and priorities. Participants emphasized that their mental health had been negatively and severely impacted by stressful life transitions and expressed pessimism about the future. They characterized a complex role played by technology, with both negative and positive effects on mental health. Technology, especially social media, could contribute to mental health issues, but the internet was also seen as a potential avenue for seeking support and reducing stigma among people of their generation. Given their regular use of smartphones and texting, participants were receptive toward automated SMS text messaging programs for mental health self-management. They appreciated that such programs provide a low-burden way to access support without the need to disclose their mental health issues to others, such as parents. Participants recommended that DMHIs include content relevant to the challenges of late adolescent life transitions (eg, high school to college, high school to workforce, and familial dependence to independence) and voiced desires for game-like engagement features and opportunities to communicate with peers. Scalable digital mental health tools, such as Small Steps SMS, may help to reduce the mental health treatment gap for adolescents if designed to meet adolescents’ unique needs.

## Appendix

Multimedia Appendix 1 Online discussion group prompts used to collect feedback from older adolescent participants on digital mental health intervention design.

## Abbreviations

ARC: asynchronous remote community
DMHI: digital mental health intervention

## Data Availability

The datasets generated or analyzed during this study are not publicly available due to the sensitive nature of text-based data and the potential risk of reidentification, but deidentified data are available from the corresponding author on reasonable request.
