# Supplementary material for: Designing Digital Mental Health Interventions to Meet the Needs of Older Adolescents: Qualitative Interview and Group Discussion Study
Source: JMIR Form Res. 2025 Aug 29;9:e68950. doi: 10.2196/68950 (PMC12432472; doi:10.2196/68950)
Supplement: Multimedia Appendix 1 [file formative_v9i1e68950_app1.docx]

**Session 1: Technologies**

To get started, let’s get to know each other by chatting about technologies we use every day and those that are our favorites. This could be things like emailing, texting, listening to podcasts, blogging, using social media, browsing the internet, watching videos or shows, or playing video games.

What technologies are your favorite to use and why?

How can technology, especially those that are your favorites, be used to help people feel less sad, down, empty, or anxious? Or, if you don’t think technology can be used to help people feel less sad, down, empty, or anxious, why do you think that is?

**Session 2: Feeling down, depressed, or empty**

It is very common to feel sad, depressed, empty, or anxious, but these feelings are different for everyone. Some mostly feel down. Others don’t feel like doing many things and don’t have their normal energy, some replay moments over and over in their head, some feel frustrated and stressed, some have problems at work and in their relationships, and most feel some combination of these things.

Which of these thoughts, feelings, and experiences are the worst and/or the hardest on you?

What is the biggest challenge in your life these days, that makes you feel down, depressed, or empty?

**Session 3: Mental wellbeing for young people**

How do you think mental health issues are different for people your age compared to people who are older?

How should support (like therapy, support groups, counseling, mental health apps and tools, etc.) look different for people your age versus for older people?

**Session 4: Text Messaging**

We want to know how you feel about receiving text messages to help deal with sadness or depression, anxiety, or other mental health concerns. You should have been receiving messages for about 3 days. **If you have not received text messages, please email or call [Research Assistant Name] at [contact details].** Please answer the following questions about your initial impressions of the Small Steps program.

Did anything surprise you about the experience of receiving messages? If so, what?

What did you like best about the experience of receiving messages, and why?

Was there anything that disappointed you or didn’t meet your expectations about the experience of receiving messages, or that needs improvement?

**Session 5: Types of Messages**

You should now have been receiving messages for about 6 days. These messages have covered a variety of topics and have taken different forms. Some days, you might get background information on skills to help with sadness and anxiety (like gratitude, positive activities, or daily rhythms), and then a suggestion for how to try that skill out in real life. Some days, you might get stories from other people. Sometimes you might be asked to write messages for other people, or receive messages written by others.

Based on your experiences so far, what types of messages have you found most interesting, and why? Which (if any) do you think might be most helpful to you in dealing with mental health concerns like sadness and anxiety?

Were there any types of messages that didn’t work very well for you, or that you think we should change?

Are there other things we could be doing with text messaging to make the program more enjoyable to use or helpful?

**Session 6: Making Small Steps Work for Younger People**

In an earlier session, we talked about how mental health issues might be different for people in your age group, or how different sorts of support might be needed. This might mean that programs like Small Steps should work differently to meet your needs. We are curious what we can do to make this program engaging and helpful for people in your age group.

What would people your age like about or be interested in about a program like Small Steps? What would people your age not like about or not be interested in about a program like Small Steps?

Are there changes we can make so that Small Steps would be more engaging or more helpful to people your age? This might mean changing the way the program interacts with you or responds to you, adding new content, changing when messages are sent, or anything else. What do you think is most important to focus on?

What would make people in your age group likely to sign up to use a program like this?

**Session 7: Stories in Small Steps**

If we were to include stories by and for people your age in Small Steps, what types of stories would be most helpful? What sorts of situations should they describe?

Give an example of a story about how you’ve taken a step to deal with feelings like being depressed, down, empty, or anxious. It’s ok if you didn’t completely resolve the problem; you can write about a time when you made some progress, or took a step that helped you feel a little better. You can write as though you were keeping a diary or writing to a close friend. Note: please do not use any full names or other identifying details in your stories. You can change the names of people or places you mention (e.g., "My friend John").

**Session 8: Other Design Directions**

Let’s say you are going to design a technology-based tool for teens to help with stress, depression, or anxiety. What would that tool look like? What features would this tool have? Your tool can use text messaging, or you can propose something completely different (online program, email service, website, app, robot, social media, game, virtual reality, etc.).
